# Cost-effective affinity support for the rapid separation of bacteria from complex food matrices

**DOI:** 10.3389/fmicb.2025.1682301

**Published:** 2025-11-25

**Authors:** Cheryl M. Armstrong, Joseph A. Capobianco, Andrew G. Gehring

**Affiliations:** United States Department of Agriculture, Agriculture Research Service, Eastern Regional Research Center, Wyndmoor, PA, United States

**Keywords:** immunomagnetic separation, Dynabeads, cell concentration, food safety, sample preparation, pathogen isolation, immunomagnetic particles

## Abstract

Advancements in molecular biology have facilitated the ability to detect microbes of interest in low abundance within complex samples. Although these technologies are extremely powerful, they typically accommodate only very small volumes of liquid samples as inputs; making sample volume a critical constraint for many molecular methodologies. Because testing volumes are often restricted to the microliter range, methods that concentrate target microbes can broaden the applicability of these detection devices. Immunomagnetic separation (IMS) is an example of a sample preparation method capable of selectively concentrating targets; utilizing magnetically-sensitive materials coated with biorecognition elements to isolate targets of interest. While the commercial availability of micron-sized, conjugation-ready, superparamagnetic particles has amplified the success of IMS for interrogating samples <10 mL, querying of large sample volumes with these particles is often financially restrictive if performed routinely. Therefore, a cost-effective alternative that can be employed for large-volume samples is presented. Here, a low-cost coating allows the conjugation of antibodies to the surface of inexpensive permanent magnets; ultimately creating an economical solid support for the selective capture of microorganisms in both buffer and ground beef homogenate (a complex food matrix). The broad utility of this method was further demonstrated by capturing either *E. coli* O157:H7 or *Salmonella enterica* through a simple antibody substitution. Novel techniques aimed at releasing target cells from the magnet via UV-light were also investigated, although the results were not definitive. Overall, expansion of IMS to large-volume food samples using this simple and economical solution could transform downstream detection capabilities in diagnostic applications.

## 1 Introduction

Food safety regulations require that microbial pathogens in food or on processing equipment in food manufacturing facilities be detected even when very low levels of pathogens are present [as low as a single cell for certain bacteria including *Escherichia coli* O157:H7 and *Listeria monocytogenes* (Archer, 2018)] in samples with relatively large masses (e.g., 325 g of beef) (United States Department of Agriculture, F. S. I. S, 2024). Although there are food diagnostics available that can accurately detect low numbers of microorganisms (Fung, 2007; Goldenberg, 2017; Rajapaksha et al., 2019; Ricke et al., 2018; Wu et al., 2017), these techniques typically accommodate the input of only very small volumes of liquid samples. This makes sample volume currently one of the biggest constraints for downstream applications, especially for tools employing molecular methods, since these techniques often restrict sample volumes used for testing to the microliter range. In food safety, culture enrichment is routinely employed because it serves to increase a pathogen’s concentration, allowing the detection threshold to be met within the small sample volumes that are utilized with detection methods such as PCR. Unfortunately, culture enrichment is a timely biological process (often taking 1–2 days) so technologies that circumvent this need are desired.

An alternative to culture enrichment is pathogen concentration, which can occur via the removal of other substances present, particularly bulk solvent. Several technologies are currently available for bacterial concentration, including centrifugation (Bisha et al., 2011; Gao et al., 2024; Kim and Oh, 2020; Solovchuk et al., 2023) and filtration-based approaches (Brewster, 2009; Kim et al., 2023; Murakami, 2012; Vibbert et al., 2015). One of the most commercially successful concentration methods not only achieves rapid separation of bulk liquid and sample matrix from targeted microbial analyte, but also selectively concentrates the target. This technology is termed immunomagnetic separation (IMS) and is based on the premise of using magnetically sensitive materials coated with biorecognition elements for the separation and isolation of targets of interest from other components within a mixture (Wang et al., 2025; Wang et al., 2020).

In general, magnetic materials can be classified into categories based upon their susceptibility to magnetic fields. Ferromagnetic and ferrimagnetic materials have a high positive magnetic susceptibility and remanence, while paramagnetic materials have a relatively low positive magnetic susceptibility and lose their magnetic properties upon the removal of an external magnetic field. Superparamagnetic iron oxide nanoparticles (SPIONs) have been coated with biorecognition elements, such as antibodies, and commercialized for the selective separation of components from complex mixtures (Kraft et al., 2017). Though SPIONs have compositions common to permanent magnets, due to their small size they only display magnetic properties when subjected to an external magnetic field, similar to paramagnetic materials (Dulinska-Litewka et al., 2019). This unique combination of short magnetic relaxation times and higher magnetic susceptibility allows the particles to be dispersed throughout a solution or suspension so that they can freely interact with targets without the influence of inter-magnetic forces yet be collected in a highly efficient manner upon the application of an external magnetic field. Several commercialized systems such as the magnetic activated cell sorting (MACS) column (United States Department of Agriculture, F. S. I. S, 2014), KingFisher™ (Chen et al., 2014), or Pathatrix™ systems (Wu et al., 2004) have been used in conjunction with food samples to enhance the capture efficiency of these particles by either increasing the ability to capture the nanoparticles or recirculating the matrix to increase the frequency of target/bead collisions, respectively.

A novel alternative to magnetic nanoparticles was devised that substantially improved the efficiency of reisolating particles from large-volume samples post processing by using only a single, macroscopic particle (Armstrong et al., 2024). This process employed a Pyrex spinbar that had been coated with antibodies for target capture through methods for conjugating antibodies to glass surfaces. The antibody-coated Pyrex spinbars were shown to successfully capture both bacterial cells and protein in sample volumes of 500 mL.

Although effective, the main drawback to all of these technologies is their expense. Typical costs without the addition of biorecognition elements range from ~$13 per test for superparamagnetic nanoparticles (with each test consisting of sample volumes around 1 mL) and ~$16 for a Pyrex spinbar, which can query sample volumes ≥500 mL. This can be cost-prohibitive for the routine testing of food samples, especially when considering that (1) current food safety protocols require the addition of a liquid medium to solid state samples such as ground beef (975 mL) or surface sampling swabs (50 mL) before being queried for the presence of microorganisms (United States Department of Agriculture, F. S. I. S, 2024) and (2) to avoid cross-contamination amongst samples, the reuse of testing components such as the superparamagnetic nanoparticles or Pyrex spinbars would not be advised.

To make IMS affordable for the routine testing of food samples, a cost-effective alternative is presented. Here, cylindrical neodymium iron boron (NdFeB) magnets, which cost under $1, were coated with antibodies and served as a solid support for the selective capture of microorganisms. These antibody-coated permanent magnets were shown to be effective at capturing *E. coli* O157:H7 in both buffer and ground beef homogenate (a complex matrix). In addition, the platform capability of this technique was demonstrated via the capture of *Salmonella enterica* and comparisons were made between the outcomes of IMS using the newly-described innovative affinity support versus commercially available superparamagnetic microparticles coated with anti-bacteria antibodies.

## 2 Materials and methods

### 2.1 Surface functionalization and antibody conjugation of NdFeB magnets

In this study, two different organosilane coupling agents were evaluated for their ability to coat neodymium iron boron magnets (Applied Magnets, Plano, TX) and included (3-Aminopropyl)triethoxysilane (APTES) and (3-Mercaptopropyl)trimethoxysilane (MPTMS). Due to the well-established methods for conjugation to glass (Chen et al., 2011; Chrisey et al., 1996; Huang et al., 2009; Warner et al., 1989; Wu et al., 2009), a Pyrex^®^ encapsulated spinbar was chosen as a control. Both the glass encapsulated spinbar (6.35 mm × 22.225 mm) and the NdFeB magnets (6.35 mm × 25.4 mm) had cylindrical geometries and similar surface areas, 5.1×10–4 m^2^(glass) and 5.7×10–4 m^2^ (NdFeB magnet). Prior to applying the coating chemistry, both the NdFeB magnets and glass encapsulated spinbar were thoroughly cleaned. To clean, the magnets were first submerged in acetone and sonicated using a Branson 2510 bath (Danbury, CT) for 60 min. The magnets were subsequently rinsed three times with Nanopure water. Piranha solution was prepared by mixing 3 parts ACS reagent grade sulfuric acid (Millipore Sigma, Burlington, MA) and 1 part 30% hydrogen peroxide (Millipore Sigma). Magnets were soaked in freshly prepared piranha solution for 15 s, rinsed three times with Nanopure water, and then dried at 100 °C for 3 h using a UNOX drying oven (Vigodarzere, Italy). Freshly cleaned magnet surfaces were coated with silane groups containing reactive groups for antibody conjugation.

For the APTES coating, the clean surfaces of the NdFeB magnets were submerged in a 2% solution of APTES in acetone for 45 s. Next, the magnets were rinsed well with acetone and dried at 150 °C for 24 h. Once dried, antibodies were conjugated to the surface using carbodiimide chemistry (Vashist, 2012). A 1 mg/mL solution of the BacTrace phosphatase-labeled affinity purified antibody to *Escherichia coli* O157:H7 (SeraCare; Milford MA), which contained an alkaline phosphatase to antibody ratio of 3 to 1, was activated with 10 molar excess 1-Ethyl-3-(3-dimethylaminopropyl) carbodiimide (EDC) and 25 molar excess N-hydroxysulfosuccinimide (Sulfo-NHS) (ThermoFisher Scientific, Waltham, MA) for 15 min, with the excess EDC/NHS subsequently removed using a Zeba Spin Desalting Column, 7 K MWCO (ThermoFisher Scientific). The APTES-coated substrate was then submerged in the activated antibody solution for 1 h and finally rinsed with phosphate-buffered saline containing 0.05% Tween-20 (PBST) (Millipore Sigma; St. Louis, MO). The PBST rinsing buffer was prepared by dissolving 1 tablet provided by the manufacturer in 1 L of Nanopure water. A final rinse was performed immediately before use in 10 mM phosphate-buffered saline, pH 7.4 (PBS).

For the MPTMS coating, a procedure previously developed for microcantilever sensors was employed (Capobianco et al., 2007). Briefly, MPTMS was deposited by submerging the NdFeB magnets in a 0.1 mM solution of MPTMS in anhydrous ethanol for 30 min and then allowing them to air dry. The dried magnets were subsequently immersed for 2 h in a 1% MPTMS solution in ethanol titrated to a pH of 4.5 using glacial acetic acid. The surface was rinsed twice with Nanopure water and dried at 150 °C for 24 h. For NdFeB magnets coated 2×, the dried magnets were immersed in freshly prepared 1% MPTMS solution in ethanol (pH = 4.5 titrated with acetic acid) for 2 h, rinsed, and dried as described above. A 1 mg/mL solution of the BacTrace phosphatase-labeled affinity purified antibody to *E. coli* O157:H7, which contained an alkaline phosphatase to antibody ratio of 3 to 1, was activated with 20 molar excess sulfosuccinimidyl 4-(N-maleimidomethyl)cyclohexane-1-carboxylate (Sulfo-SMCC) for 30 min and excess SMCC was removed using a Zeba Spin Desalting Column, 7 K MWCO (ThermoFisher Scientific). The MPTMS coated substrate was then submerged in the activated antibody solution for 2 h and finally rinsed with PBST. Antibody-coated NdFeB magnets were stored at 4 °C in PBST until use. Immediately before use, the antibody-coated NdFeB magnets were warmed to room temperature and a final rinse was performed in PBS.

### 2.2 Colorimetric assay for the quantitation of antibody along the surface of the NdFeB magnets

Each magnet to be assayed was placed into a 1.5 mL centrifuge tube and 600 μL of a 1 mg/mL p-Nitrophenyl phosphate (pNPP) solution in 0.2 M Tris (prepared from the manufacturer’s tablet) was added to each tube as a substrate for the reaction. Tubes were incubated at room temperature for 20 min in a dark environment, with the magnets being removed from the solution to stop the reaction. The amount of colored product resulting from the conversion of the pNPP substrate by the alkaline phosphatase conjugated to the antibodies was measured via the absorbance of a 100 μL aliquot of the reaction at 405 nm using a Safire^2^ plate reader (Tecan Group Ltd.; Männedorf, Switzerland). If the initial sample was too concentrated (absorbance > 0.4), the sample was diluted 10-fold in Nanopure water and the measurement was repeated. Two of each type of magnet were cleaned but not coated with silane for use as controls.

### 2.3 Capture of *Escherichia coli* by the antibody-coated NdFeB magnets using different spin rates

A culture of *E. coli* O157:H7-PC (Paoli et al., 2015) was grown overnight at 37 °C with agitation in Luria Bertani (LB) broth (BD Difco; Franklin Lakes, NJ) containing 100 μg/mL of spectinomycin (Sigma; St. Louis, MO). The next day a 1:100,000 dilution of the overnight culture was made in 0.1% Buffered Peptone Water (BPW), of which 35 mL was dispensed into individual sterile 100 mm × 15 mm polystyrene petri plates for experimentation and a 10 μL sample was plated in duplicate onto LB agar containing 100 μg/mL of spectinomycin to obtain accurate cell counts for the inoculum. An anti-*E. coli* antibody-coated NdFeB magnet (3.175 mm × 12.7 mm) was placed into the plates containing the bacterial culture and stirred using a typical laboratory stir plate for 10 min at 100, 200, 350, or 500 rpm. The transfer of the antibody-coated NdFeB magnets was achieved using another magnet to facilitate their movement. A silane-coated NdFeB magnet that did not contain antibodies was used as a negative control. A single wash of the magnet was performed by transferring the magnet to a clean petri plate containing 35 mL PBS (pH 7.1) and stirring for 2 min to remove any loosely bound material. The magnets were then transferred to sterile individual 0.2 mL PCR tubes and 150 μL of nuclease free water was added to completely cover the magnet. Tubes were boiled at 100 °C for 10 min and then cooled to 4 °C in a T100 Thermal cycler (BioRad; Hercules, CA) to lyse the captured cells. The magnets were subsequently removed and the tubes were spun at 10,000 × g for 5 min at 4 °C to remove cell debris with the supernatant being placed into a clean tube. From there, 8 μL of the supernatant was placed into a MicroAmp Fast Reaction tube (Applied Biosystems; Foster City, CA) along with 10 μL of Dynamo Flash master mix (ThermoFisher Scientific), 0.5 μL of STEC-Shuffle-F primer (20 μM), 0.5 μL of STEC-Shuffle-R primer (20 μM), 0.25 μL of STEC-Shuffle-P probe (20 μM), 0.4 μL of 50X ROX, and 0.35 μL of dH_2_O. (This primer/probe combination amplifies a specific genomic marker within the *E. coli* O157:H7-PC strain and allows it to be differentiated from *E. coli* strains naturally present in the matrix.) Quantitative real-time PCR (qPCR) was performed using conditions identical to those reported by Paoli et al. (2015) to determine cell capture.

### 2.4 Effects of time on the capture of *Escherichia coli* by the antibody-coated NdFeB magnets

A culture of *E. coli* O157:H7-PC was grown overnight at 37 °C with agitation in LB broth containing 100 μg/mL of spectinomycin. The next day a 1:100,000 dilution of the overnight was made in 0.1% BPW to yield a solution of 1 × 10^4^ CFU/mL. Thirty-five mL of this solution was dispensed into individual sterile 100 mm × 15 mm polystyrene petri plates for experimentation and a 10 μL sample was plated in duplicate onto LB agar containing 100 μg/mL of spectinomycin to obtain the inoculum cell counts. An anti-*E. coli* antibody-coated NdFeB magnet (3.175 mm × 12.7 mm) was placed into the plates containing the bacterial culture and stirred using a typical laboratory stir plate for 1, 5, 10, or 20 min at 350 rpm. The magnet was then washed for 2 min, the captured cells lysed, and qPCR was performed using the STEC-Shuffle primer/probe set as described above. Once again, a silane-coated NdFeB magnet that did not contain antibodies was used as the negative control.

### 2.5 Conjugation and testing of anti-*Salmonella* antibodies on the surface of the silane coated NdFeB magnets

The surface of the NdFeB magnets was conjugated with either anti-*E. coli* or BacTrace anti-*Salmonella* common structural antigen (CSA)-Plus antibodies (SeraCare) using the conjugation protocol specific for MPTMS as described above. The resulting antibody-coated magnets were placed in either 30 mL of an *E. coli* O157:H7-PC or 35 mL of a *Salmonella enterica* subspecies *enterica* serovar Minnesota K^+^ (Armstrong et al., 2019) culture diluted 1:10,000 or 1:100,000. In addition, a 10 μL sample from each dilution was plated in duplicate onto LB agar containing either 100 μg/mL of spectinomycin or 40 μg/mL of kanamycin to obtain accurate cell counts for the inoculum. The antibody-coated NdFeB magnet was stirred for 10 min at 350 rpm and washed for 2 min before lysing the captured cells. Resulting DNA was used to amplify a genetic marker via qPCR in both strains with either the STEC-Shuffle-F / STEC-Shuffle-R /STEC-Shuffle-P primer/ probe set or the Sal-dnaE-Shuffle-F (TCGCTACTTCCTGGAACTGATC) + Sal-dnaE-Shuffle-R (CCATTACTGCATGGCACAACTC) + Sal-dnaE-Shuffle-Probe (TGGAATACGTCTTCGTCTCTGCTCAGTAGC) using the conditions set forth by Paoli et al. (2015). Silane-coated NdFeB magnets that did not contain antibodies served as negative controls. Three independent trials were conducted with *E. coli* and four using *S. enterica*.

### 2.6 Antibody conjugation of superparamagnetic beads

Tosylactivated M-280 Dynabeads (Invitrogen; Waltham, MA) were conjugated with BacTrace anti-*E. coli* O157:H7 antibody (SeraCare) using the guidelines provided in the product technical data sheet. First, the bead suspension (provided by the manufacturer in purified water) was resuspended using a vortex for 1 min, sonicated in a Branson 2510 bath (Danbury, CT) for 20 min, and vortexed again for 1 min. A 333 μL aliquot of the suspension (10 mg of Dynabeads) was transferred to a 1.5 mL centrifuge tube. One mL of 0.1 M sodium phosphate buffer, pH 7.4 [2.62 g NaH_2_PO_4_ × H_2_O (MW 137.99) and 14.42 g Na_2_HPO_4_ × 2 H_2_O (MW 177.99); adjusted to 1 L with Nanopure water] was added to the centrifuge tube and vortexed for 1 min. Using the DynaMag 2 magnet (Invitrogen), a magnetic field was applied to one side of the tube for 3 min to collect the beads, and the supernatant was discarded. The processed was repeated two additional cycles and then the beads were resuspended in 200 μL of 1 mg/mL solution of antibody. The suggested protocol states the optimal coupling bead concentration is 40 mg/mL, and the coupling solution should contain 1.2 M ammonium sulfate. To achieve these criteria, 50 μL of 6 M ammonium sulphate in sodium phosphate buffer (79.284 g of ammonium sulphate was added to 100 mL of 0.1 M sodium phosphate buffer and heated to 70 °C to facilitate dissolution) was added to the bead suspension in 200 μL of 1 mg/mL antibody solution (total reaction volume = 250 μL). The suspension was vortexed for 10 s and then incubated on a tube rotator (Dynal Biotech Inc.; Milwaukee, WI) at 37 °C for 20 h. Using the DynaMag 2 magnet, a magnetic field was applied for 3 min, and the supernatant was discarded. One mL of 0.5 wt% bovine serum albumin (Sigma-Aldrich; St. Louis, MO) in PBS, pH 7.4 (Millipore-Sigma) is added to the centrifuge tube, vortexed for 10 s, and incubated on a tube rotator at 37 °C for 1 h. The beads were washed using the DynaMag 2 magnet, by applying a magnetic field for 3 min and discarding the supernatant. One mL of 0.1 wt% bovine serum albumin (Sigma-Aldrich) in PBS, pH 7.4 (Millipore-Sigma) was added to the centrifuge tube, vortexed for 10 s and the supernatant was removed using the DynaMag 2 magnet for 3 min. To ensure a 20 mg/mL final bead concentration, the beads were resuspended in 0.5 mL of 0.1 wt% bovine serum albumin in PBS. For the control beads, the antibody was substituted with bovine serum albumin.

### 2.7 Testing of superparamagnetic beads and NdFeB magnets in buffer and ground beef homogenate

Testing to compare the antibody-conjugated superparamagnetic beads to the antibody-conjugated NdFeB magnets was performed on actively dividing cells to maximize the capture of live cells versus cell fragments that do not contain DNA on the beads/magnets. Here, cultures of *E. coli* O157:H7-PC were grown to mid-log phase in LB broth containing 100 μg/mL of spectinomycin at 37 °C with agitation. The culture was diluted 1:10,000 in 0.1% BPW to yield ~4 × 10^3^ CFU/mL and 30 mL aliquots were dispensed into 50 mL conical tubes (Fisher Scientific; Waltham, MA) for experimentation while a 10 μL sample was plated in duplicate onto LB agar containing 100 μg/mL of spectinomycin to obtain inoculum cell counts. One 3.175 mm × 12.7 mm antibody-coated magnet (coated with MPTMS 2x) or 6 μL of the prepared M-280 Tosylactivated Dynabeads were added to the 50 mL conical tubes containing *E. coli* and the tubes were subsequently allowed to rotate 360° on a tube rotator (Fisher Scientific) for 10 min. These conditions minimized the experimental variables, by making the surface area of the beads equivalent to that of the NdFeB magnet. The NdFeB magnets were washed in clean conical tubes containing 30 mL of sterile PBS (pH 7.1) for 2 min while the Dynabeads were washed by allowing 10 mL of 0.1 M sodium phosphate buffer, pH 7.4, followed by 2 mL of 0.5 wt% bovine serum albumin in PBS, pH 7.4 to flow across the beads while the beads were suspended within a MACS large cell separation column (Miltenyi Biotec; Bergisch Gladbach, Germany) in contact with a strong separation magnet. The Dynabeads were eluted from the column with 150 μL of nuclease free water while the NdFeB magnets were transferred to 0.2 mL PCR tubes containing 150 μL of nuclease free water. Lysis of captured cells on both the Dynabeads and the NdFeB magnets was performed via the boiling method and cell debris removed by centrifugation. Supernatant (8 μL) was utilized for qPCR with the STEC-Shuffle primer/probe set described above. Three independent trials of each type of assay were conducted. Identical analyses were performed in ground beef homogenate prepared by stomaching 114 g of 80–20 ground beef purchased from a local supermarket in 350 mL BPW except the culture was diluted 1:1,000 in 0.1% BPW to yield ~5 × 10^4^ CFU/mL. Two independent trials of each type of assay were conducted.

### 2.8 Addition of a photocleavable linkage for release of cells from the NdFeB magnets

Antibodies that were attached to a biotinylated oligonucleotide that also contained a photocleavable linkage were conjugated to the surface of the MPTMS coated NdFeB magnets (see section 2.1 above for production procedures for the MPTMS coated NdFeB magnets). For this, a 5 mg/mL solution of the NeutrAvidin, was activated with 20 molar excess sulfosuccinimidyl 4-(N-maleimidomethyl)cyclohexane-1-carboxylate (Sulfo-SMCC) for 30 min and excess SMCC was removed using a Zeba Spin Desalting Column, 7 K MWCO (ThermoFisher Scientific). MPTMS coated NdFeB magnets were then submerged in the activated NeutrAvidin solution for 2 h and finally rinsed with PBST. BacTrace anti-*E. coli* O157:H7 antibodies (SeraCare) were provided to Bio-Synthesis Inc. who constructed a short poly T oligo containing the conjugated anti-*E. coli* O157:H7 antibody, a photocleavable linker, and a dual biotin modification, ultimately producing the following product: [Antibody]-[azide-DBCO linkage]-[PC linker]-[5′-TTTTT-3′]-[biotin]-[biotin] (Bio-Synthesis Inc., Lewisville, TX). The NeutrAvidin coated NdFeB magnets were then allowed to incubate in the biotinylated oligo solution for 2 h and rinsed with PBST before use.

Assays were performed as stated previously using magnets containing the photocleavable oligo/antibody constructs described above in a 35 mL suspension of *E. coli* at ~5 × 10^4^ CFU/mL in LB broth with a spin rate of 350 rpm and an exposure time of 10 min prior to the wash. Post-washing, magnets were placed in sterile 0.2 mL PCR tubes and covered with 120 μL of PBS. For cleavage of the linkage attaching the cells to the magnet to occur, the capped PCR tubes containing the magnets and PBS were exposed for 5 min to near-UV light using the Mineralight model UVGL-58 (Ultra-Violet Prod., Inc., San Gabriel, CA) long wavelength (365 nm) at a distance of 11 cm. These parameters were used in an effort to achieve the near-UV light wavelengths and time necessary for complete cleavage as stated by the manufacturer. The tubes containing the magnets were turned 3–4 times throughout the 5 min interval to increase exposure of the different sides of the magnet to the light. Post-exposure, the total volume of PBS within the tube was dispensed across 3 LB agar plates containing 100 μg/mL of spectinomycin. The magnet was placed into 1 mL of LB broth to help establish if viable bacterial cells remained attached to the magnet post-cleavage. Both the LB plates and broth tubes were incubated overnight at 37 °C and CFUs were recorded for the plate assays while turbidity was noted for the liquid media. In total, 7 independent trials were analyzed. Coated NdFeB magnets that were not exposed to UV light and NdFeB magnets that were not coated with the oligo/antibody constructs were used as controls for cleavage and binding, respectively.

### 2.9 Statistical analysis of results

Statistical processing and data presentation was carried out using the JMP software version 14. Student *t*-tests were conducted under the assumption of approximate normality and equal variances to evaluate if the differences seen between the means for the various methods of antibody attachment and other parameters related to cell capture by the particles were statistically significant. The level of significance was set at 0.05 with different letter designations demarking samples that were found to be significantly different.

## 3 Results

### 3.1 Application for antibody-coated NdFeB magnets

**Figure 1:**
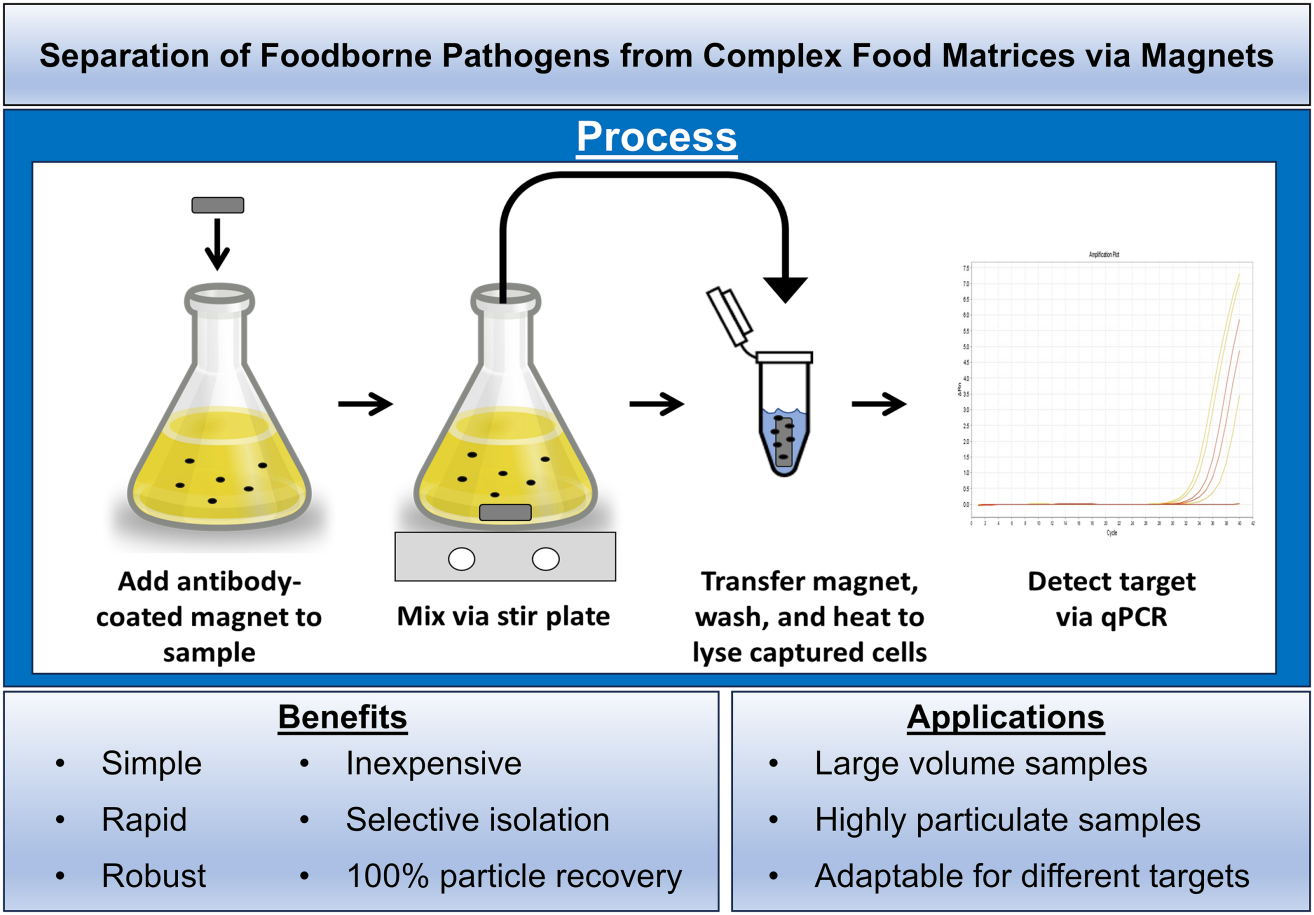
Schematic outlining the process and utility for using antibody-coated neodymium iron boron magnets to concentrate bacteria from complex mixtures.

There are several different types of immunomagnetic particles currently on the market and numerous manufacturers of these particles. Regardless of the type of immunomagnetic particle or the manufacturer, the commercially available products share some common characteristics; namely their paramagnetic properties, their small sizes (all within the micrometer/nanometer size range) and their application for the effective separation of various biological materials in small volume samples. However, their small size limits their utility for the routine testing of large sample volumes because of the expense associated with the number of beads within that size range theoretically needed to interrogate volumes greater than 10 milliliters. To overcome this limitation, antibody-coated NdFeB magnets >1 centimeter in length were designed so that large volume samples could be queried in an economical fashion (Figure 1). The large size of the NdFeB magnets not only allows for a simple yet effective mixing strategy when combined with a magnetic stir plate but also aids in their retrieval, resulting in 100% recovery compared to nano-sized immunomagnetic particles that can be lost during sample manipulation.

### 3.2 Surface functionalization of the NdFeB magnets

**Figure 2:**
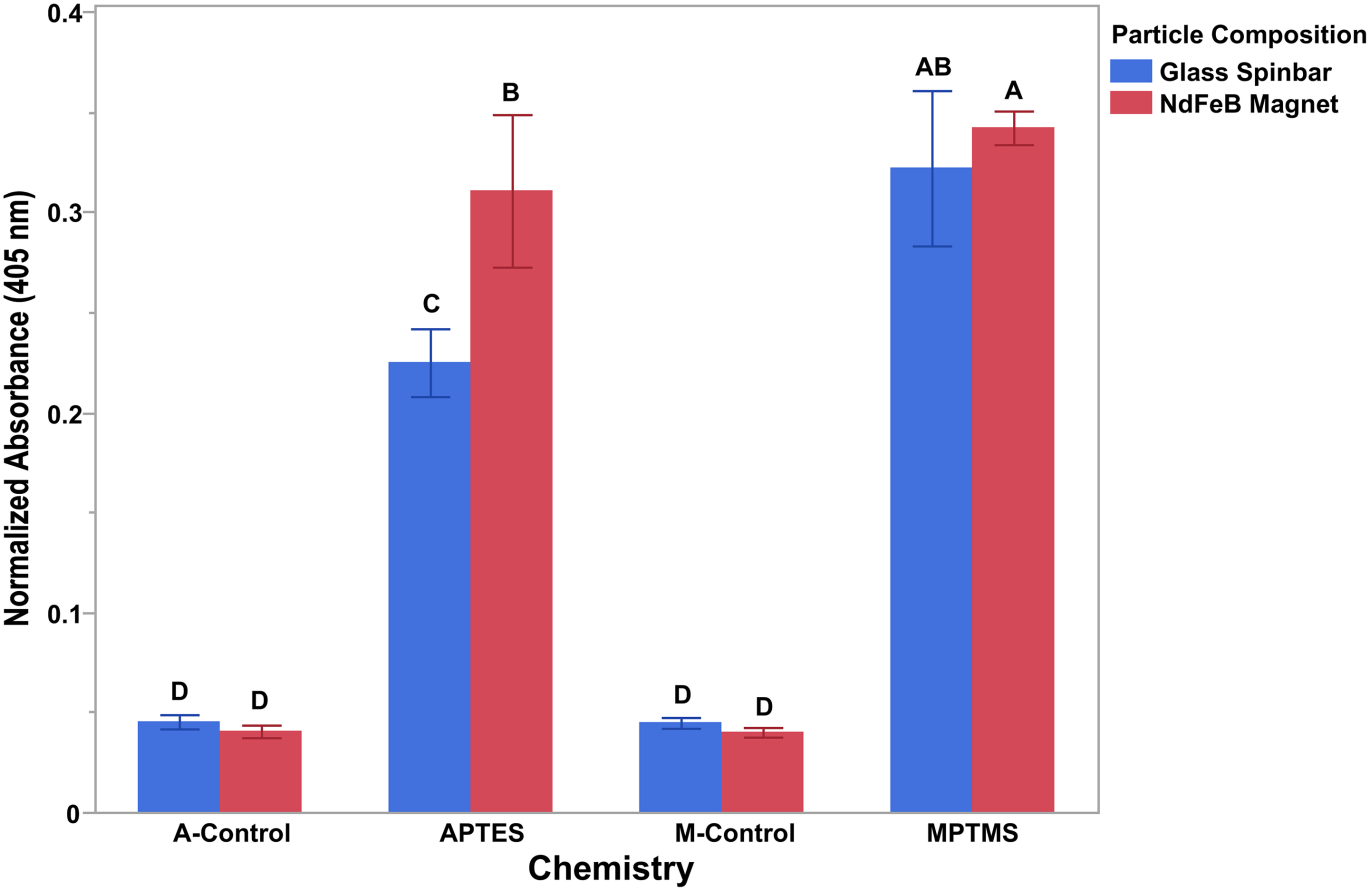
Antibody attachment on both glass and neodymium iron boron when amine and sulfhydryl (mercapto) chemistries were used to functionalize the surfaces. The conversion of p-Nitrophenyl phosphate to colored p-Nitrophenol by an alkaline phosphatase-conjugated antibody demonstrated the ability of both the amine (APTES) and the sulfhydryl (MPTMS) chemistries to functionalize the surface allowing for the conjugation of antibodies to the surface of both glass-coated stir bars and NdFeB magnets. APTES (A-) and MPTMS (M-) controls consisted of cleaned magnets not coated with silane. Individual *t*-tests were conducted between all presented groups with differing letters denoting significance and error bars representing the standard deviation of the mean.

Proper surface coating is a key component for the effective conjugation of antibodies to any surface. Although conjugation schemes exist for the deposition of antibodies onto a variety of substances, none describe conjugation of antibodies to the surface of a NdFeB magnet. Because of this, a silane coupling agent was employed to first coat the surface of the NdFeB magnet, allowing for the addition of an organic functional group (amine, sulfhydryl, epoxy, etc.) onto which a biorecognition element could be conjugated. Two different organosilane coupling agents were evaluated in this study for their coating ability and included (3-Aminopropyl)triethoxysilane and (3-Mercaptopropyl)trimethoxysilane. Given the establishment of methods for conjugating antibodies to glass, a Pyrex® spinbar was chosen as a control for these experiments (Armstrong et al., 2024). To determine the coating efficiency, a colorimetric assay was conducted on both the coated NdFeB magnets and the Pyrex® glass encapsulated spinbar (Figure 2). For this assay, alkaline phosphatase conjugated anti-*E. coli* O157 antibodies were deposited onto the surface of the NdFeB magnets treated with MPTMS, APTES, and the Pyrex® spinbar so that the alkaline phosphatase present on the antibody would produce a color change when in the presence of an appropriate substrate measurable by optical density (OD). This allowed the amount of antibody on the magnet to be determined semi-quantitatively. Because the surface area of the glass and magnet surfaces differed slightly (5.1×10^−4^ vs. 5.7×10^−4^ m^2^), which can affect the amount of conjugated antibody, the measured OD was normalized by the surface area to directly compare the relative effectiveness of the conjugation strategies. As the surface area of the glass magnets was smaller, the OD measured for these materials was multiplied by 1.12, which is the ratio of the (NdFeB) magnet surface area divided by the surface area of the glass-coated magnet (the OD for the Nd magnets was multiplied by 1, its surface area divided by its surface area). To simplify the *x*-axis, the conditions are grouped by magnet, where the blue bars represent data collected for the glass spinbars and the red bars for NdFeB magnets. Although graphically, the data is grouped by magnet, all data was treated independently. Independent *t*-tests were conducted between all groups presented, with different letters denoting groups that are statistically different from one another. Of the four non-coated controls, none were found to be different from one another (*p* > 0.76) while all controls were statistically different from the experimental groups (*p* < 0.001), which indicated that antibodies were indeed conjugated to the surfaces. While the antibody-conjugated surface using the amine coating on glass was statistically different from the controls, it was also statistically different from the NdFeB magnet antibody-coated surface (*p* < 0.0001). Conversely, antibody-conjugated surfaces using the mercapto/sulfhydryl chemistry did not differ significantly when glass versus an NdFeB magnet was used (*p* = 0.081). However, differences were significant when comparing the mercapto/sulfhydryl chemistry on the NdFeB magnet to the amine chemistry on the NdFeB magnet (*p* = 0.0031), with the NdFeB magnet also displaying a greater amount of antibody attachment compared to the glass. Overall, both the sulfhydryl and the amine chemistries appeared to effectively facilitate the conjugation of antibodies to the surface of both Pyrex spinbars and NdFeB magnets. However, because the mean observed in trials utilizing MPTMS was slightly higher, combined with the fact that MPTMS provided a more consistent and practical coating method across different substrates, all subsequent trials employed MPTMS as the organosilane coupling agent for the NdFeB magnets.

### 3.3 Parameters for optimal cell capture using antibody-coated NdFeB magnets

Once conjugation of the antibodies to the surface of the magnet was shown to be successful, several additional parameters were investigated to determine the optimal conditions for cell capture with the antibody-coated NdFeB magnets. The first parameter optimized was the rate of spin of the antibody-coated magnets for bacterial capture. The optimal stir rate for the antibody-coated magnets was determined by evaluating cell capture at spin rates of 100, 200, 350, or 500 rpm (Supplementary Figure 1A). Results from qPCR assays demonstrated that cell capture was highest at 350 rpm.

In addition to the rate of spin, the length of time the antibody-coated NdFeB magnets were in contact with the sample was also optimized so that highest amount of cell capture could be achieved in the shortest amount of time possible (Supplementary Figure 1B). Optimal exposure time for the antibody-coated magnets was determined by evaluating cell capture at 1, 5, 10, or 20 min. Capture efficiency appeared to increase with exposure time up to 10 min, after which no further gains were observed.

### 3.4 Specificity of the magnet was determined by the biorecognition elements

**Figure 3:**
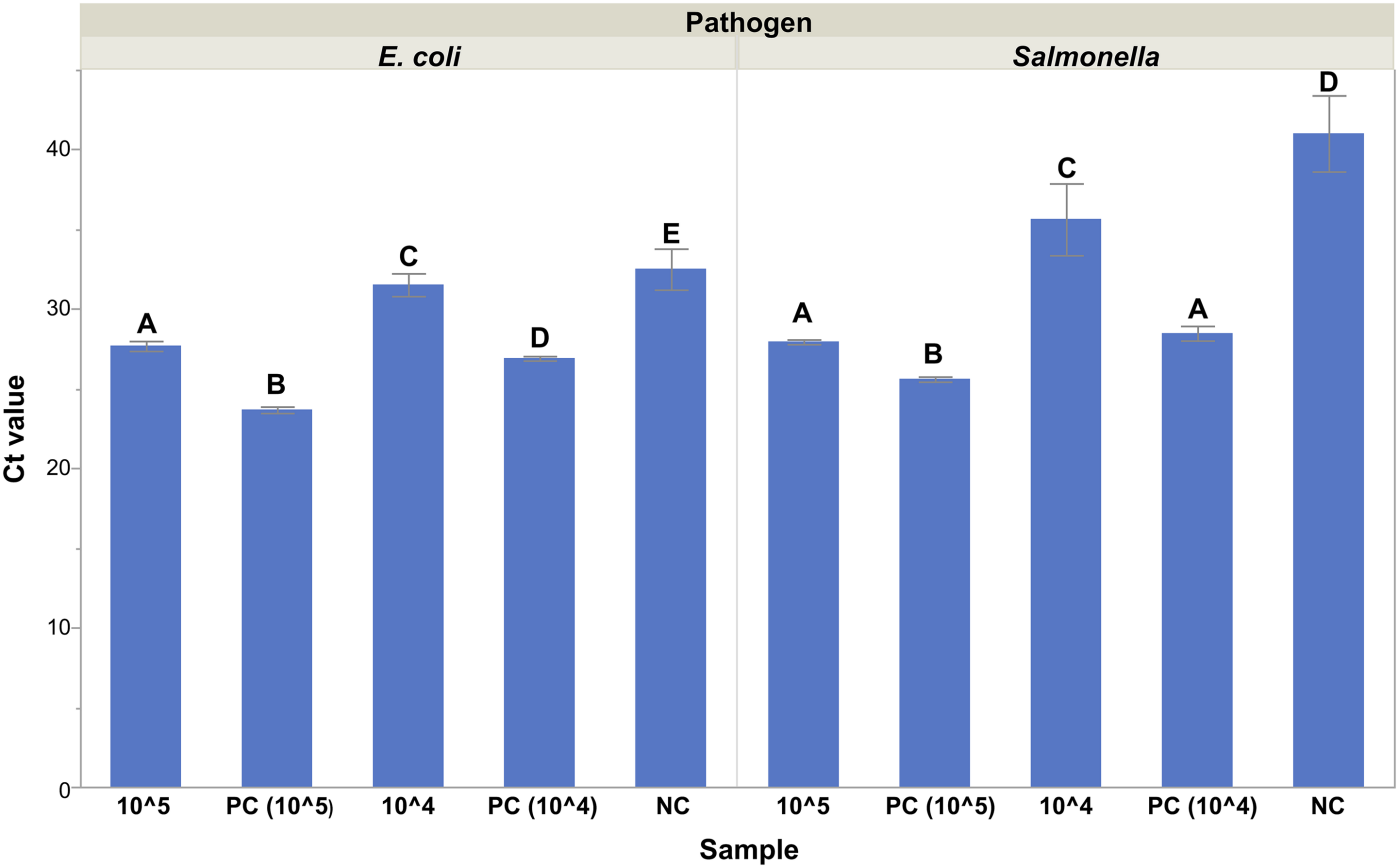
Biorecognition elements determine the specificity of the magnets. Magnets conjugated with either anti-*E. coli* or anti-*Salmonella* antibodies were mixed with two different concentrations (10^4^ or 10^5^ CFU/mL) of *E. coli* (left) or *S. enterica* (right), respectively. Ct values representing the number of cells captured by the antibody-coated NdFeB magnets were measured via qPCR with primers/probes specific to the cells of interest. Student’s *t*-tests were performed independently for each pathogen tested. Error bars represent the standard deviation of the mean. Silane coated NdFeB magnets that did not contain antibodies but were exposed to 10^5^ CFU/mL of cells were used as negative controls (NC) while an aliquot of the 10^4^ or 10^5^ CFU/mL cultures served as the positive controls (PC).

To validate the ability of the magnet to function as a platform technology and capture different cell types, either anti-*E. coli* antibodies or anti-*Salmonella* antibodies were conjugated to the NdFeB magnets. The antibody-coated NdFeB magnets were then subjected to two different concentrations (10^4^ or 10^5^) of either *E. coli* or *S. enterica* depending upon the antibodies used and Ct values, representative of the number of cells captured, were measured via qPCR (Figure 3). Student’s *t*-tests were performed for each of the two matrices tested to determine the ability of the antibody-coated NdFeB magnets to capture the cell types of interest. From this, it was demonstrated that the NdFeB magnets could capture different targets and that the capture of various targets of interest could be achieved by simply exchanging the biorecognition element on the NdFeB magnets.

### 3.5 Influence of the amount of silane coating on cell capture

**Figure 4:**
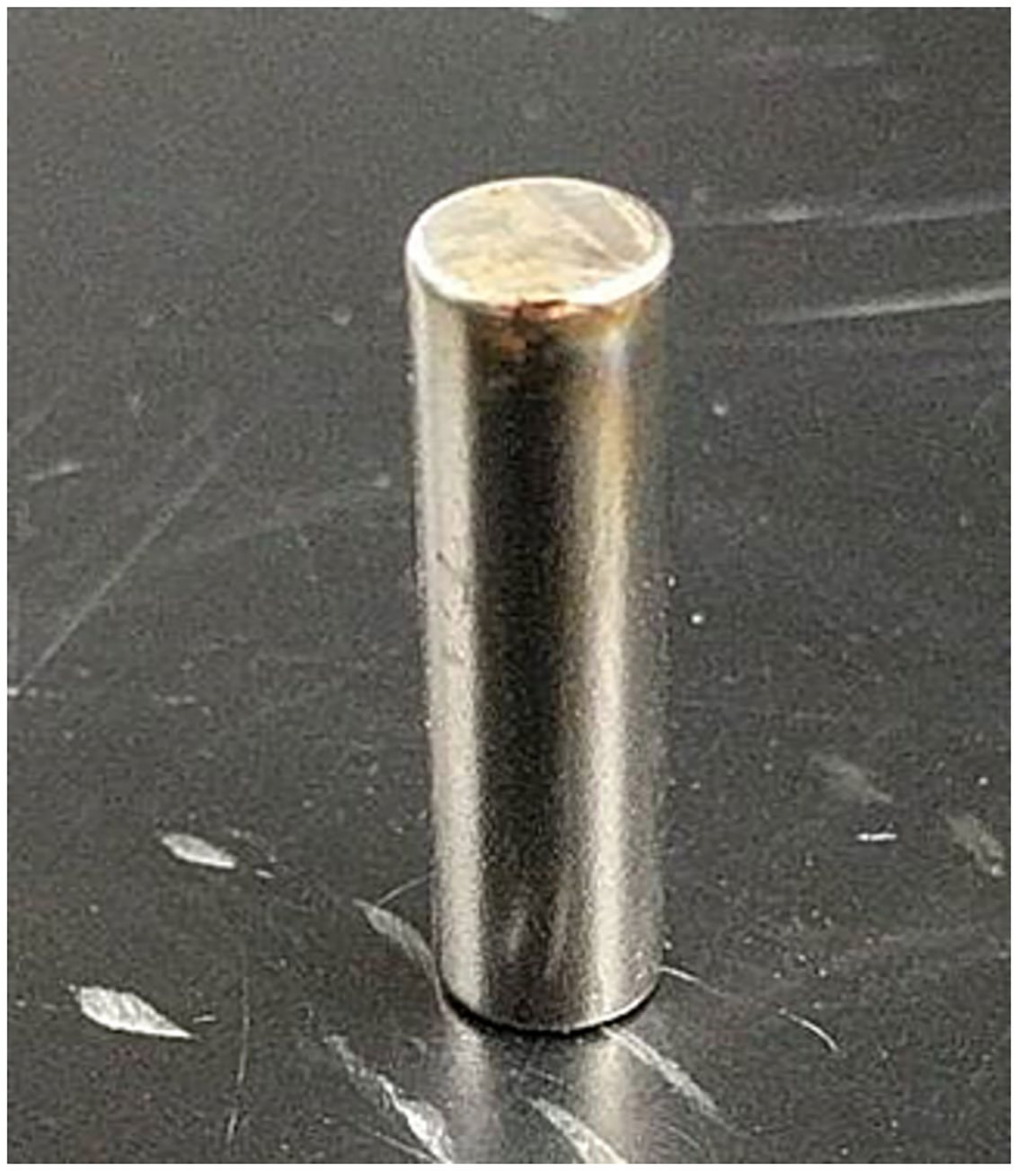
Corrosion present on the neodymium magnets. The storage conditions of the antibody-coated NdFeB magnets (4 °C in PBST) appeared to elicit corrosion as noted on the upper surface of the magnet shown.

**Figure 5:**
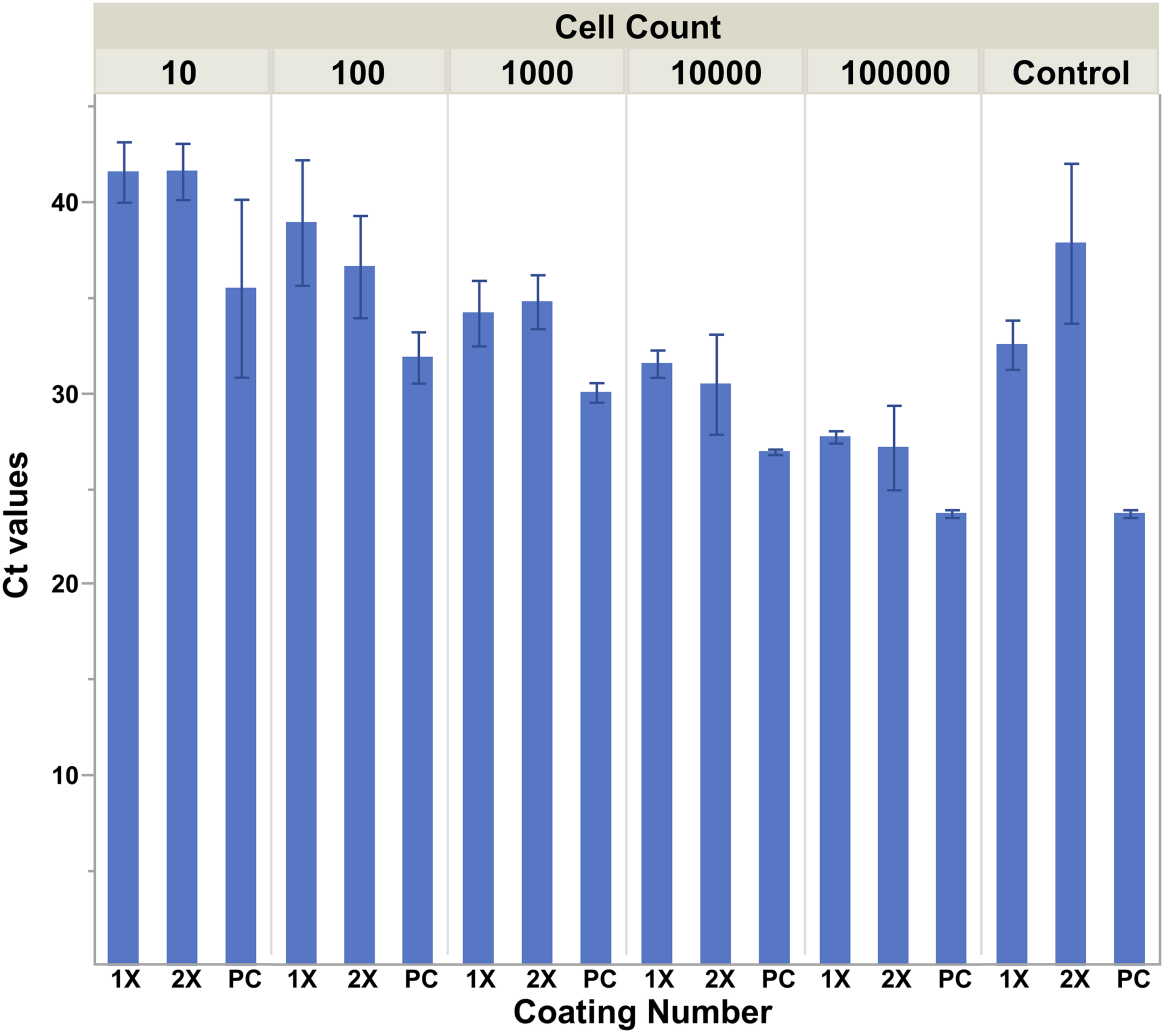
Cell capture is influenced by the amount of silane coating on the magnets. NdFeB magnets were prepared for conjugation by coating them with silane via a 2 h incubation in a 1% solution of (3-Mercaptopropyl)trimethoxysilane either once (1×) or twice (2×). Post coating, anti-*E. coli* antibodies were conjugated to the magnets, which were then exposed to a 30 mL solution containing ~10^5^, 10^4^, 10^3^, 10^2^, or 10^1^ CFU/mL of *E. coli* cells. The capture ability of the magnets was determined by qPCR using primers/probe specific for the cells of interest. Averages are plotted with error bars representing the standard deviation of the mean.

Upon storage of the antibody-coated NdFeB magnets at 4 °C in PBST, it was noted that some magnets began to corrode (Figure 4). In an effort to reduce the potential for corrosion, NdFeB magnets were prepared for conjugation by coating them with silane either once (1x) or twice (2x). Post coating, anti-*E. coli* antibodies were conjugated to the magnets, which were then subjected to a solution containing ~ 10^5^, 10^4^, 10^3^, 10^2^, or 10^1^ CFU/mL of *E. coli* cells. Coating of the NdFeB magnets twice with MPTMS appeared to increase capture since there is a slight drop in the mean Ct value, although the difference was not statistically (Figure 5). It is also noteworthy that significantly more non-specific capture was observed using the no-antibody NdFeB magnet controls with only a single coating compared to controls that were coated twice, thus highlighting the role of the coating in minimizing non-specific binding. Not only did the 2×-coated magnets yield a higher signal-to-noise ratio compared to their 1×-coated counterparts but the additional coating also appeared to visually diminish post-storage corrosion.

### 3.6 Comparison of superparamagnetic beads to NdFeB magnets for the capture of *Escherichia coli* O157:H7

**Figure 6:**
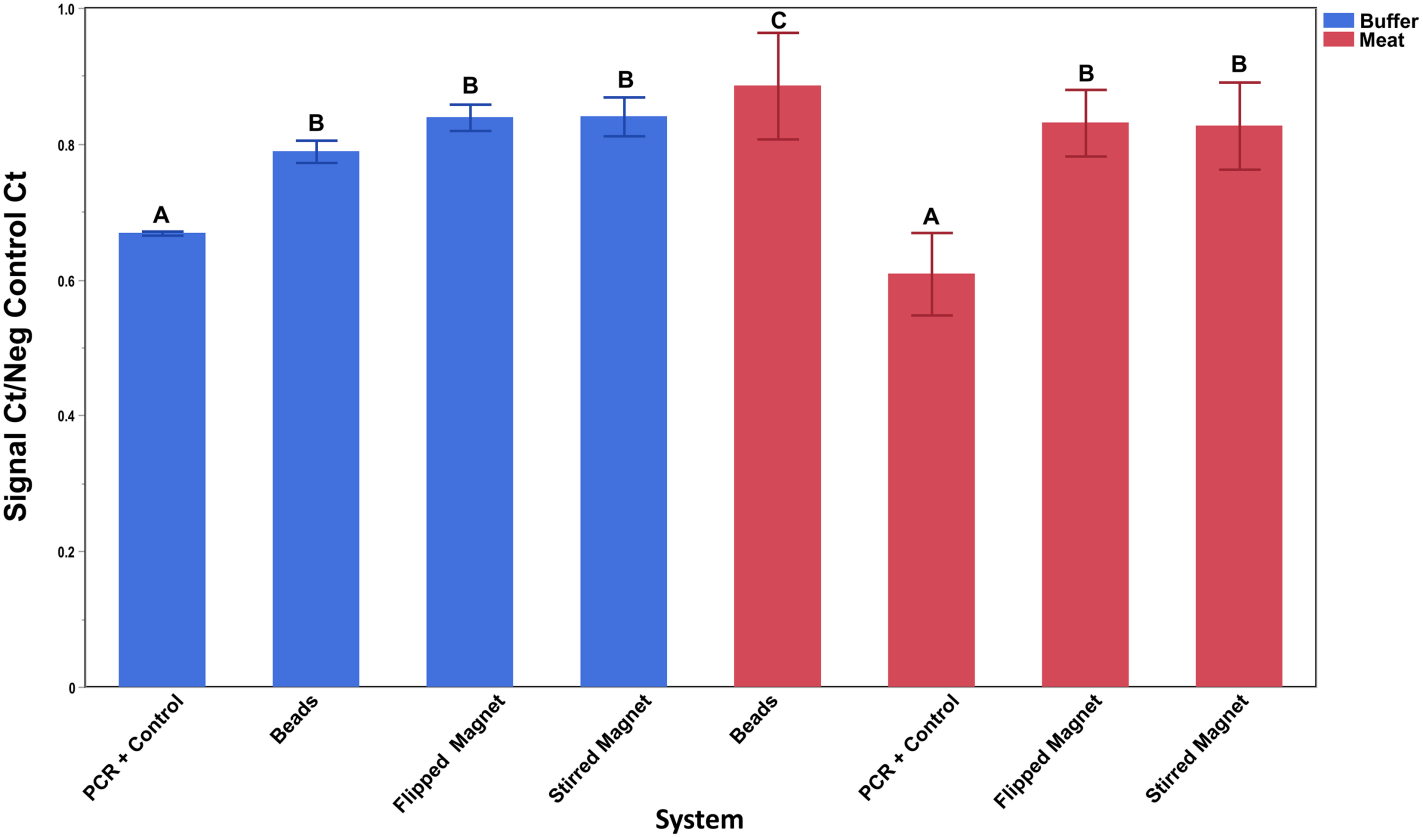
Capture of *E. coli* O157:H7 by antibody-conjugated superparamagnetic beads and antibody-conjugated NdFeB magnets. Silane-coated NdFeB magnets and commercially available superparamagnetic beads were both conjugated with anti-*E. coli* antibodies before exposure to either a 0.1% PBW solution (blue bars) or a ground beef homogenate (red bars) containing ~4 × 10^3^ CFU/mL or ~5 × 10^4^ CFU/mL of *E. coli* cells, respectively. The capture ability of the beads and the NdFeB magnets was determined by qPCR using primers/probe specific for the cells of interest. Student’s *t*-test was performed to determine significance and error bars denote the standard deviation of the mean.

Capture by the antibody-conjugated NdFeB magnets compared to commercially available antibody-conjugated superparamagnetic Dynabeads was conducted in both inoculated BPW and a complex food matrix (ground beef homogenates) (Figure 6). Because the manufacturer’s protocol for the Dynabeads recommended that mixing occur using a tube rotator, both the beads and one set of magnets (flipped bar) were handled in this fashion. Since antibody-conjugated NdFeB magnets also have the unique ability to be mixed with a sample via a stir plate, this condition was also tested during this assay (stirred bar). (No attempt was made to mix the beads using the stir plate since the magnet in the base of the stir plate simply pulled the beads out of suspension.) The washing process for the beads differed from the bar as noted in the materials and methods since several initial attempts without the use of the MACS large cell separation column resulted in a non-detectable PCR signal from the Dynabeads. Results from this assay demonstrated the following: (1) cell capture by both the antibody-conjugated Dynabeads and the antibody-conjugated NdFeB magnets was similar in buffer (*p* ≥ 0.06) while (2) NdFeB magnets recovered significantly more cells than the Dynabeads from the ground beef homogenate (*p* ≤ 0.001). It is also worth noting that both the recovery and washing processes are not only simpler but also quicker for the NdFeB magnets when compared to the beads.

### 3.7 Removal of captured target from the NdFeB magnets

To further the utility of the NdFeB magnets for use as a sample preparation technique, a method was devised to detach the captured cells from the magnet. Proof-of-principle experiments were conducted aimed at releasing the captured cells from the magnet. For this, anti-*E.coli* antibodies were attached to a biotinylated oligonucleotide containing a photocleavable linkage with the oligonucleotide undergoing subsequent conjugation to the surface of a NeutrAvidin-coated NdFeB magnet. This design allows targeted release of antibody–cell complexes from the magnetic support since cleavage occurs at the photo-cleavable site upon UV exposure, separating the antibody from the biotin moiety that anchors it to the surface. Cell capture was performed as previously described and cells were cleaved from the NdFeB magnet through a 5-min exposure to a long-wavelength near-UV light source. The resulting cell suspension was spread plated onto media and bacterial growth was recorded as colony forming units (CFUs) (Supplementary Figure 2). Based upon the quantification of CFUs, cells could be cleaved from the NdFeB magnet, although it should be noted that the process did not appear to be particularly effective. In support of this statement is the fact that magnets that were not directly exposed to the UV light source still showed the release of cells from the magnets in three of the seven trials, resulting in a large standard deviation (189 ± 259 CFU/mL). In addition, after processing, each magnet was placed into media to test for growth of cells that remained attached to the magnet. In five of the seven trials performed, the anti-body coated magnets showed growth in the culture media post-exposure to the UV light source indicating that removal of the cells from the magnets was not complete. Further refinements to the protocol are necessary to address these issues so that a more robust cleavage procedure can be attained.

## 4 Discussion

Cell capture by immunomagnetic particles is defined in part by both mass transport and collision probability, whereby increasing the number of particle-cell interactions should result in a concomitant increase in the number of cells captured (Irwin et al., 2002; Tu et al., 2003). Because of this, effective mixing is essential to achieving optimal cell capture with IMS due to its role in maximizing the number particle-cell interactions that occur. Instead of attempting to disperse the particles within the sample to improve particle-cell interactions, as is done with superparamagnetic nanoparticles, the current method takes a different approach by establishing turbulent flow via a laboratory stir plate (Armstrong et al., 2024; Critchley, 2017) and drawing the sample down to the magnetic particle through the creation of a vortex. Although both antibody-coated NdFeB magnets as well as Pyrex spinbars take advantage of this and produce a device capable of interrogating large sample volumes with high collision probabilities for capturing targets of interest, NdFeB magnets can do it at a much lower cost. Pyrex spinbars typically range in price from $16 to $20 while NdFeB magnets of a similar size range from $0.15 to $0.81 apiece. The low cost enables their application for routine food safety screening methods, since the need to avoid cross-contamination drives the use of disposable equipment within the industry. Reagents used for the silane-coating of the NdFeB magnets were also inexpensive on a per magnet basis, keeping the total cost below $1, and not only facilitated the covalent conjugation of the selective biorecognition elements but also served as a corrosion barrier.

The integrity of the corrosion barrier can be important because surface defects such as scratches can accelerate corrosion, which shortens the effective shelf life of the antibody-conjugated NdFeB magnets. The corrosion initially observed on some of the magnets was consistent with observations commonly associated with pitting corrosion as the material erosion was concentrated near physical defects such as scratches (Figure 4). Corrosion can affect the effective surface area of the magnet and the number of biorecognition elements that can participate in the capture of a target. Furthermore, ions from the inner layers of the magnet may leech into the elution solution and possibly interfere with downstream detection platforms such as PCR. Therefore, optimization of the surface treatment through the application of additional silane to the magnet not only provided protection against corrosion but also allowed efficient deposition of coupling agents that serve as linkage sites for conjugation to selective molecules, ultimately enhancing the reliability and repeatability of the magnets for cell capture.

Findings from this study are consistent with our earlier research and further demonstrate the effectiveness of a single macroscopic-sized magnet for target capture especially in heterogenous food samples, likely stemming from factors such as decreased matrix interference as well as increased particle recovery and/or reduced entrapment of the magnet by the matrix as discussed previously (Armstrong et al., 2024). It is important that the diminished performance of the Dynabeads in the ground beef homogenate be interpreted within the context of the experimental design, noting that the limited Dynabead volume and differences in washing methods may have also played a role in their recovery efficiency. Nevertheless, because the performance of the Dynabeads was essentially equivalent to that of the NdFeB magnets in buffer, the change in matrix may be more critical compared to the differences in the wash methods between the two groups.

Additional strategies proven to enhance the capture efficiency of other devices by optimizing packing density (such as those aimed at separating the particles from the substrate or orienting the biorecognition elements) could also be applied to the NdFeB magnets to further increase capture rates. For example, Guo et al. (1994) evaluated the effect of spacer length, surface density, and hybridization conditions on oligonucleotide hybridization and established the importance of the spacer length for antibody–antigen interactions. They demonstrated that the extra length provided by a spacer decreased steric hindrance while increasing flexibility, ultimately allowing immobilized ligands to move in positions that establish the correct binding orientation with target proteins. Of the numerous polymeric spacer arms available, polyethylene glycol (PEG) has been used to successfully increase the capture of antigen by immobilized antibodies (Soltys and Etzel, 2000). In addition to reducing steric forces, PEG has also been demonstrated to be an excellent medium for preventing nonspecific binding (Furuya et al., 2006). Therefore, future work should focus on identifying methods to optimize the packing density, separation from the substrate, and orientation of biorecognition elements on the surface to maximize target capture.

Use of the current protocol for producing antibody-coated magnets for IMS has many advantages. Aside from offering the same simple mixing strategy, 100% particle recovery, and compatibility with large-volume and highly particulate food samples as seen with Pyrex stirbars (Armstrong et al., 2024), it provides a substantial saving in material costs, which allows it to be readily integrated into established workflows. For example, immuno-concentration protocols are currently an integral part of methodologies aimed at the isolation of foodborne pathogen. These protocols are typically performed after culture enrichment, which greatly extends the time-to-results. Unfortunately, enrichment cannot be avoided given the volume constraints of the current methods for IMS. However, employment of antibody-coated magnets for IMS could be a viable substitution for magnetic nanoparticles since they reduce the time needed by either decreasing or potentially eliminating the lengthy culture enrichment step by providing a means to capture cells from large-volume samples. In addition, the ability to conjugate various antibodies to the surface of the magnets increases the range of targets that can be selectively isolated. They are also highly adaptable and can be expanded to other diagnostic tools. For instance, use of magnets made from the rare-earth metal neodymium may not be necessary. The surface functionalization protocol presented here may be applicable to other magnetic materials, which could expand its use while further reducing costs, although reductions in magnetic strength may be seen as well with alternative materials. Additionally, applicability of the method can be broadened by adjusting the size of the magnets such as incorporating micro-sized (flea) magnets into lab-on-a-disk techniques. However, the corresponding decrease in functional surface area must also be considered for applications where the capture of a large number of targets is required.

Separation of the cells from the magnet can be beneficial because it enables certain analysis to be performed that could not be performed if the cells remain attached to a macroscopic object, such as dispersing cells across agar plated media to quantify colony forming units. However, the proof-of-principle experiments aimed at releasing the cells via a photocleavable linkage were not fully successful. Not only were some cells released from the control magnets, possibly through indirect exposure to UV-light within the laboratory setting, but viable cells remained attached to the magnets post UV-exposure in a majority of the trials. Alterations to the exposure time or the UV-light intensity was not tested because of the possible detrimental effects that UV-light can have on living bacterial cells (Kowalski, 2009). Given that destructive methods are not always desirable for analysis, future studies should focus on a more reliable method for release of viable/intact cells from the magnets since this is a limitation of the current construction. Alternative techniques that disrupt the intermolecular forces between the biorecognition element and the cells such as affinity-based competitive ligand displacement (Hirsch et al., 2002), adjustments in pH (Kandimalla et al., 2004; McGovern et al., 2007), or salt concentration (Loo et al., 2011) could be used. It is also possible that commercialized kits designed for the detachment of cells from paramagnetic nanoparticles (Frenea-Robin and Marchalot, 2022) may be effective at separating the cells from the magnets, thus enhancing the overall versatility of the method for sample preparation.

In conclusion, antibody-coated NdFeB magnets represent a cost-effective strategy for the isolation of pathogens that can be implemented in complex food matrices. The simple mixing of the antibody-coated magnets with a sample for 10 min, which was performed using a common laboratory stir plate, followed by a 2 min wash step permitted the capture, separation, and isolation of the target cells of interest from the food matrix. The reduction in sample volume and elimination of non-target material allows the captured target to be analyzed by an array of downstream molecular detection devices for pathogen determination such as qPCR or enzymatic analysis for ATP production. Moreover, these antibody-coated NdFeB magnets act as a platform technology, which can be broadly applied to any number of targets for which biorecognition elements are available with the possibility of multiplexing through the addition of several different biorecognition elements to a single magnet or via the sequential application of multiple magnets coated with different antibodies to a single sample. By combining the simplicity of a single permanent magnet with its adaptability for a multitude of biorecognition elements, this method will help further the development of affordable diagnostic solutions that rely upon the isolation of cells from complex mixtures.

## Data Availability

The original contributions presented in the study are included in the article/Supplementary material, further inquiries can be directed to the corresponding author.
