# Supplementary material for: Cost-effective affinity support for the rapid separation of bacteria from complex food matrices
Source: Front Microbiol. 2025 Nov 25;16:1682301. doi: 10.3389/fmicb.2025.1682301 (PMC12685795; doi:10.3389/fmicb.2025.1682301)
Supplement: Supplementary file 1 [file Table_1.docx]

**Cost-Effective Affinity Support for the Rapid Separation of Bacteria from Complex Food Matrices**

**Supplemental Figures:**


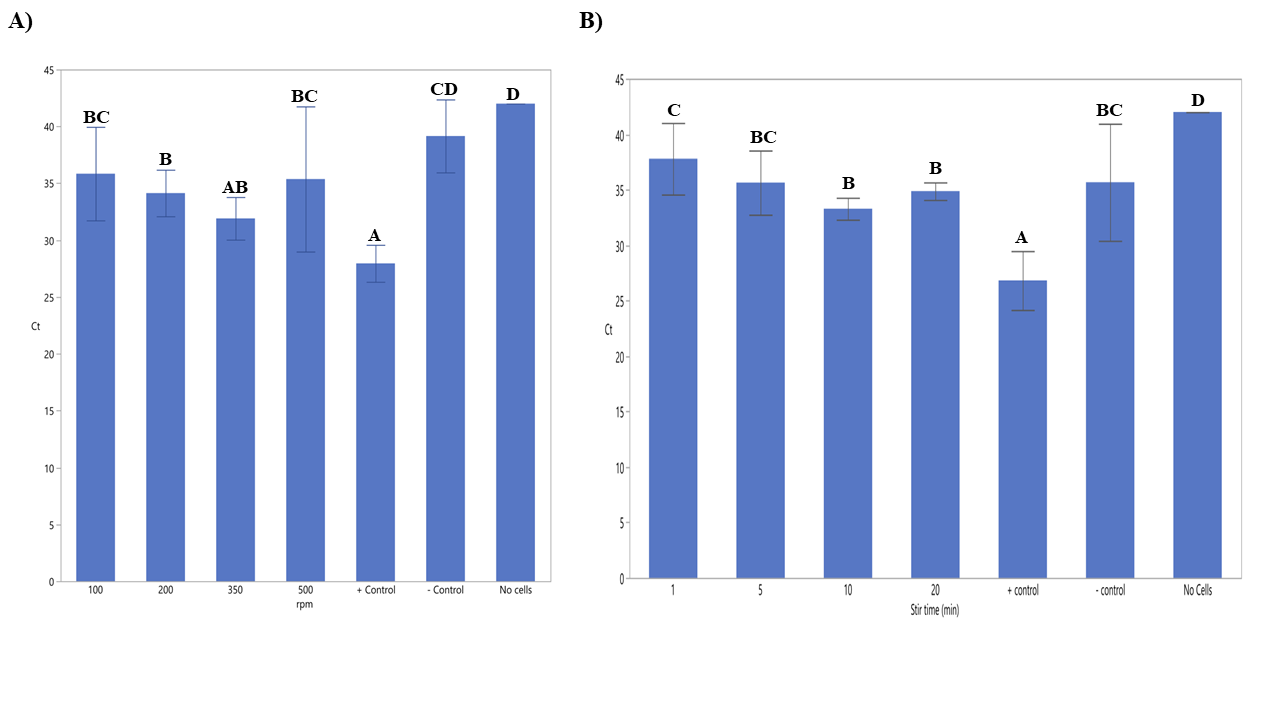


**Supplemental Figure 1: Parameters affecting cell capture by the antibody-coated magnets. A)** The optimal stir rate was determined by evaluating the ability of an anti-*E. coli* antibody-coated NdFeB magnet to capture cells when spun at a rate of 100, 200, 350, or 500 rpm within a petri dish containing 35 mL of ~1 X 10^4^ CFU/mL *E. coli*. **B)** Optimal exposure time was determined by evaluating the ability of an anti-*E. coli* antibody-coated NdFeB magnet to capture *E. coli* cells upon exposed to a 35 mL culture containing ~1 X 10^4^ CFU/mL for 1, 5, 10, or 20 min. Silane-coated NdFeB magnets not coated with antibodies were exposed to *E. coli* cultures for use as a negative control while an aliquot of the diluted culture acted as a positive control. Ct values resulting from qPCR assays were plotted to assess *E. coli* cell capture in both assays. Student’s t-tests were performed to determine significance with error bars denoting standard deviation of the mean.


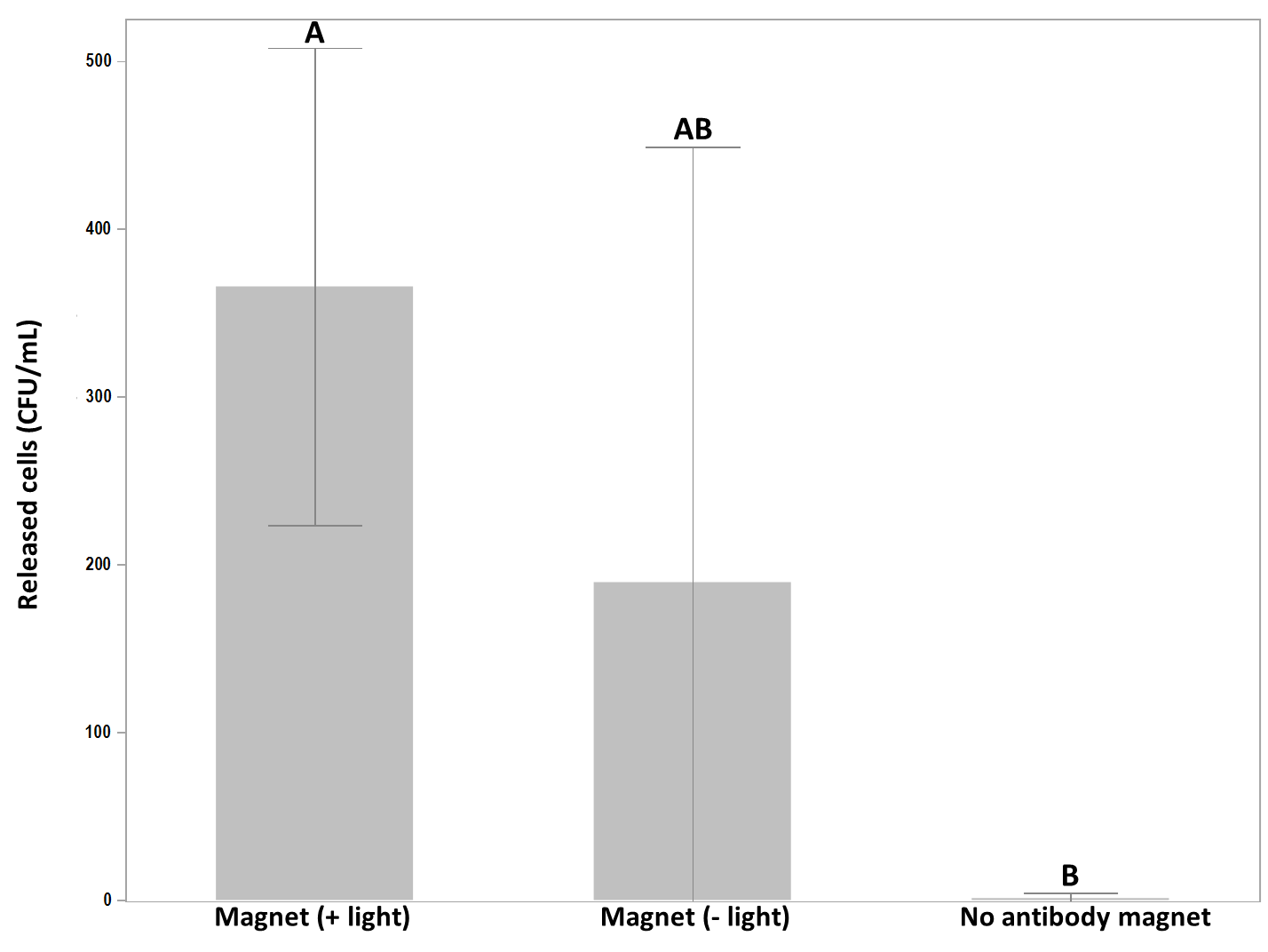


**Supplemental Figure 2: Release of captured cells from** **the magnetic capture surface.** To release captured cells from the magnets, a short poly-T oligo containing both a photocleavable linkage and an anti-E. coli antibody was attached to the magnets. Cleavage of the linker using long-wavelength UV light was tested for its ability to release the captured cells into a buffered solution surrounding the magnets. Magnets containing the poly-T oligo/antibody conjugate that were not exposed to UV light and magnets that did not contain the poly-T oligo/antibody conjugate were used as controls.
